# Dry Sliding Friction of Tool Steels and Their Comparison of Wear in Contact with ZrO_2_ and X46Cr13

**DOI:** 10.3390/ma13102359

**Published:** 2020-05-20

**Authors:** Michal Krbata, Maros Eckert, Lenka Bartosova, Igor Barenyi, Jozef Majerik, Pavol Mikuš, Petra Rendkova

**Affiliations:** 1 Faculty of Special Technology, Alexander Dubcek University of Trenčín, 911 06 Trenčín, Slovakia; michal.krbata@tnuni.sk (M.K.); lenka.bartosova@tnuni.sk (L.B.); igor.barenyi@tnuni.sk (I.B.); jozef.majerik@tnuni.sk (J.M.); pavol.mikus@tnuni.sk (P.M.); 2 Department of Political Science, Alexander Dubcek University of Trenčín, 911 06 Trenčín, Slovakia; petra.rendkova@tnuni.sk

**Keywords:** tool steel, tribology, ZrO_2_ counterpart, coefficient of friction

## Abstract

Tool steels are used in stamping, shearing processes, and as cutting tools due to their good mechanical properties. During their working cycle, steels are subject to aggressive conditions such as heat stress, fatigue, and wear. In this paper, three tool steels, namely X153CrMoV12, X37CrMoV5-1, and X45NiCrMo4 were selected against two types of bearing balls, ZrO_2_ and X46Cr1. All measurements were performed on a UMT TriboLab universal tribometric instrument under dry conditions. The main objective of the experiment was to analyze and compare tool steel wear in contact with two kinds of bearing balls with a diameter of 4.76 mm. This evaluation is focused on the hardness, surface roughness, and microstructure of all samples and on the impact of the input parameters on the resulting wear. All three types of tool steels were measured in the basic annealed state and, subsequently, in the state after hardening and tempering. Experimental results show that tool steels, belonging to high strength steels, can successfully represent wear resistant steels. The content of carbide elements, their size, and shape in the microstructure play an important role in the friction process and subsequent wear. Three types of loads were used and compared in the experiments 30, 60, and 90 N. Increasing the load results in significant degradation of the material on the sample surface. Lastly, the impact of hardness and roughness of materials on wear has also been proven. If abrasive wear occurs in the friction process, there is a greater degree of wear than that of adhesive wear. This is due to less abrasive particles, which behave like a cutting wedge and are subject to subsequent deformation strengthening due to the load increase, which adversely affects the further friction process. Analysis of the results showed that the ZrO_2_ ceramic ball showed significantly better wear values when compared to the X46Cr13 stainless steel ball. It also improves the values of the coefficient of friction with respect to the type of wear that occurs when the experimental materials and counterparts are in contact.

## 1. Introduction

Various types of tool steels are used in the manufacture of tools and components, which are subjected to high loads and wear during the friction process. Therefore, the resistance to these loads significantly affects their service life. They are most often used in milling, drilling, sawing, and measuring tools as well as in the production of various types of molds. Many scientific papers in recent years have focused on investigating the wear of several types of tool steels. Bourithis et al. [1] investigated the wear properties of two tool steels (X153CrMoV12 and O1) using the pin on the disc method. Their results clearly show that the microstructure of tool steel plays the most important role in determining the wear properties. Omer and Muammer [2] studied and compared the wear of seven different materials (X153CrMoV12, Vanadis 4, Vancron 40, K340 ISODUR, Caldie, Carmo, 0050A) using a newly developed wear test facility. The best values in this study were Vancron 40 steel, which had up to 70% less wear than X153CrMoV12 tool steel. Sen et al. [3] examined the tribological behaviour of steel in a tester that worked on the ball on disc principle. In these tests, X153CrMoV12 tool steel was used as the opposite sliding surface. The results showed that the coefficient of friction decreases as the speed of sliding increases, while the wear rate decreases. Some researchers [4,5,6] have investigated the wear behavior of X153CrMoV12 tool steel and the resistance to deep cryogenic hypothermia under different friction speeds and normal loads. They considered that the wear behaviour of tool steel can be clearly correlated with austenite content reduction and an increase in the secondary carbide particles in the microstructure. Celik et al. [7] dealt with X37CrMoV5-1 steel on which they applied different types of coatings and investigated wear at different temperatures. Cihak-Bayr et al. [8] investigated high plastically deformed sub-surface tribozones in sliding experiments on X37CrMoV5-1 steel. The main emphasis was on understanding the modification of the microstructure in the field of friction energy transformation in tool steel. The authors [9,10] dealt with the thermal treatment of the X37CrMoV5-1 tool steel surface layer to increase wear resistance. The authors’ results are focused on evaluating the surface hardness after thermal influence of the material surface and the relationship with the resulting microstructure. These results clearly indicate less wear with increasing material hardness. The tool steel X45NiCrMo4 is mostly nickel alloyed and has a lower chromium content than previous steels. Therefore, very fine contents of iron carbides can be observed in this steel and, in view of this fact, it also has a lower hardness value than the first steel examined in the ground state. Pougis et al. [11] investigated dry friction of steel under high pressure in quasi-static conditions on X45NiCrMo4 tool steel. This author is one of the few who deals with the impact of high loads (above 100 N) on wear. Their friction coefficient decreased as a function of normal contact pressure. The coefficient of friction was also directly dependent on the roughness of the contact samples. Dishliev et al. [12] also investigated the wear resistance of X45NiCrMo4 tool steel by applying a multi-layer Ti/TiN/TiCN based nanocomposite coating to this steel. From their results, it is evident that, as the load increases, the wear rate of the material increases. Knowledge of the coefficient of friction is also important in numerical simulations where general values of the coefficient of friction relative to contact materials are generally used [13,14,15,16]. However, friction depends on a number of input factors such as rate, pressure, type of material in contact with temperature, and surface roughness. Most of the authors in the papers dealing with the friction of tool steels and their wear deal mainly with one material, which is mostly the treated surface or the use of very low loads, which are not in practice.

**Figure 1:**
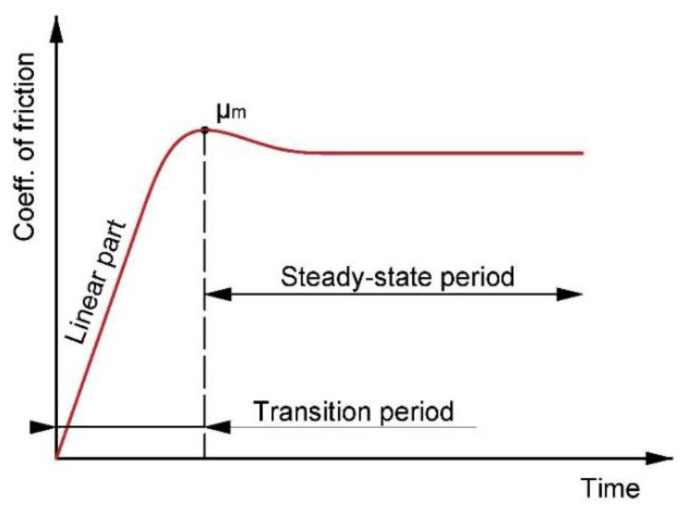
Typical friction coefficient curve.

In this work, dry sliding friction on selected tool steels and their comparison of wear in contact with ZrO_2_ and X46Cr13 bearing balls were investigated. There are many types of devices designed to measure the coefficient of friction and wear, often designed for a particular field of research such as brakes in the automotive industry [17,18]. In our case, the entire friction measurement was performed in the UMT TriboLab, where the main parameter was the load (30, 60, 90 N). The basic microstructures of the supplied tool steels were investigated using optical and electron microscopy (SEM) equipped with an energy dispersive spectroscope (EDS). The microstructure was also investigated with an atom force microscope (AFM) to obtain a high-resolution microstructure and the size and shape of the individual phases and carbides in the microstructure of the material. Additionally, using the atomic force microscope (AFM), 3D topographies of the balls used as a counterpart were obtained. The roughness of the material and the balls was also determined from the obtained 3D topographies. All obtained friction and wear coefficient results were discussed in the next part of the paper. Figure 1 shows a typical friction coefficient curve, which can be divided into two main parts. The first part is called a transition portion comprised of a run-in portion with a mostly short linear portion. At the end of this section is usually the peak of the friction coefficient. The second part of the curve is the steady-state period, where the friction coefficient curve stabilizes. Depending on the selected parameters and the types of contact pairs, the second part of the curve may have either a slightly decreasing or a rising direction [19].

## 2. Materials and Methods 

The basic materials used in these experiments were three types of tool steels. The first material is the high-alloy tool steel X153CrMoV12 used in the engineering industry. It is a high-harden chrome-vanadium steel suitable for oil and air hardening [20]. The steel is characterized by high wear resistance and very high tensile strength, mainly used for cutting tools such as profile presses and complex shape cutters. The carbon content of 1.53% has an impact on increasing the wear resistance of tool steel, especially after heat treatment. The high chromium content increases the strength and supports the formation of chromium carbides and increases the corrosion resistance. Vanadium in a given steel has a beneficial effect on grain growth and improved fatigue properties of the material. Manganese and molybdenum increase the strength in materials without deteriorating plasticity, and, in combination, increase the hardenability. 

The second material was the medium alloy tool steel X37CrMoV5-1 used for hot working. Steel is similar in chemical composition when compared to the previous one, except that the main chromium alloying element is less than half that of the previous steel. This tool steel is designed for very stressed tools such as dies, hot cutting tools, and knives [21]. Lower carbon content in steel reduces wear resistance. The increased content silicon increases the strength but deteriorates the formability of the steel. The lower chromium content compared to the previous steel reduces the size and shape of the carbides.

The last one was the medium alloyed tool steel X45NiCrMo4. This tool steel is alloyed mainly with nickel and chrome. The use of steel is cold working for all kinds of tools that are designed for stamping and forging [22]. The steel is alloyed with a high nickel content of 4.05%, which, together with chromium, softens the crystallization, increases the yield strength, and ensures higher values of toughness at normal and reduced temperatures. The chemical composition of the experimental samples was verified by the Q4 TASMAN spectral analyser and are shown in Table 1. A comparison of mechanical and physical properties of each experimental sample are listed in Table 2. 

**Figure 2:**
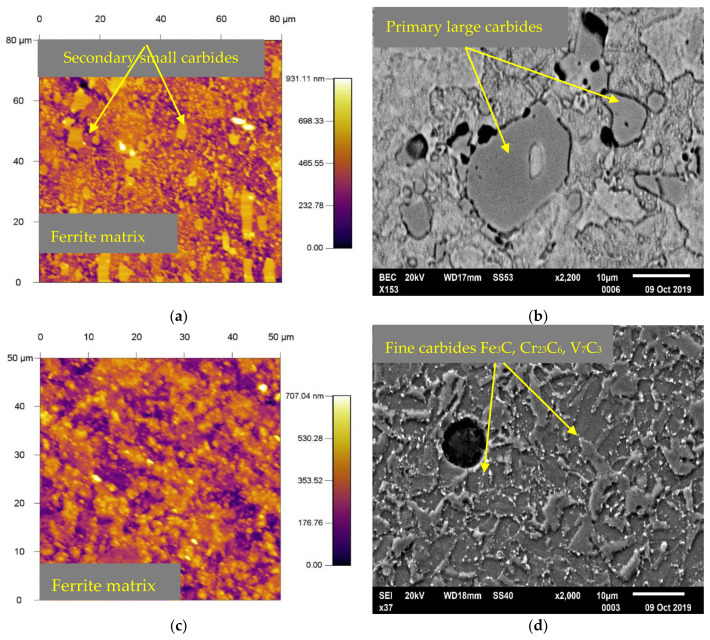

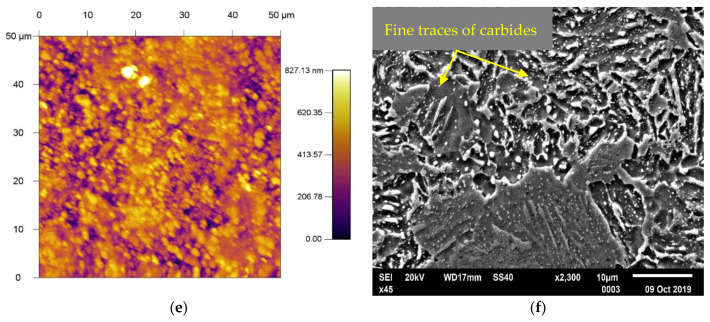
Basic microstructures of experimental tool steels (annealed). (**a**) X153CrMoV12 (by AFM), (**b**) X153CrMoV12 (by SEM), (**c**) X37CrMoV5-1 (by AFM), (**d**) X37CrMoV5-1 (by SEM), (**e**) X45NiCrMo4 (by AFM), (**f**) X45NiCrMo4 (by SEM).

**Figure 3:**
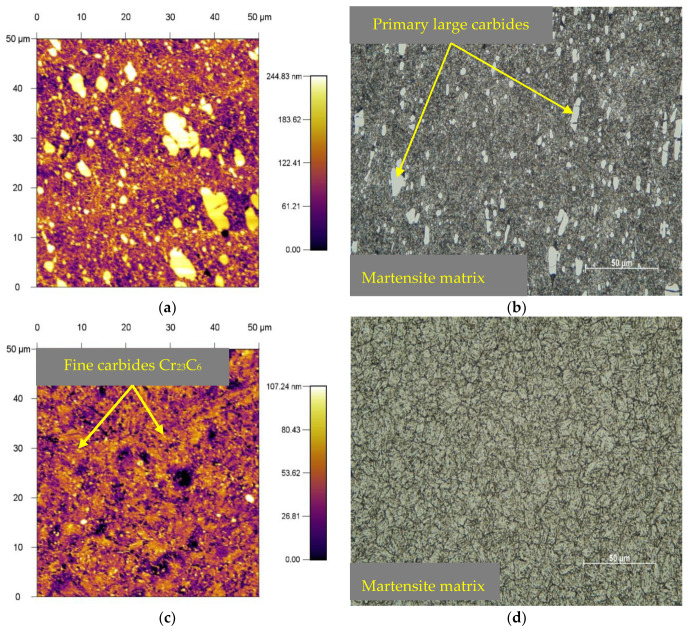

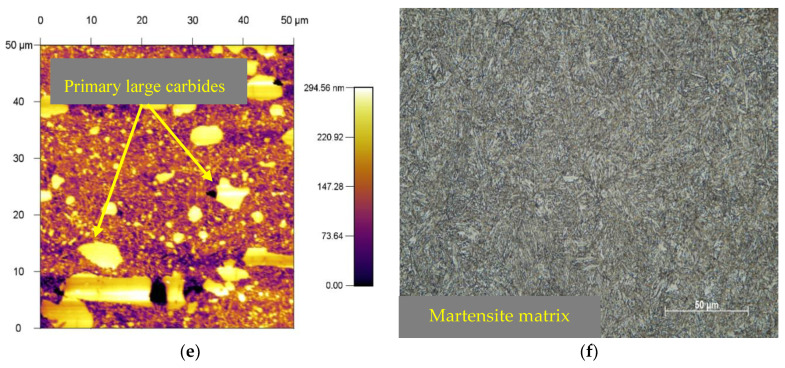
Basic microstructures of experimental tool steels (hardened and tempered), (**a**) X153CrMoV12 (by AFM), (**b**) X153CrMoV12 (by LOM), (**c**) X37CrMoV5-1 (by AFM), (**d**) X37CrMoV5-1 (by LOM), (**e**) X45NiCrMo4 (by AFM), (**f**) X45NiCrMo4 (by LOM).

The base materials were supplied in the form of bars with a diameter of 10 mm and a length of 1000 mm. The supplied material is produced in electric furnaces with the possibility of treatment of liquid steel in the units of secondary metallurgy. X153CrMoV12 was supplied in a soft annealed state with heating at 800 °C throughout the cross-section, which was followed by cooling in the furnace at a rate of 20 °C/h with a maximum hardness of 270 HV5. The microstructure of the base material showed the occurrence of coarse and fine spheroidized carbide particles in the ferrite matrix, which is highly machinable and offers less deformation resistance when compared to other microstructures produced by quenching tool steels. The larger spheroidized carbide particles shown in Figure 2a,b are the primary carbides of M_7_C_3_ formed during solidification, which were dispersed as a result of the heat treatment. The finer carbides are derived from secondary precipitation in carbide spherodization, which results from the transformation of austenite to the ferrite carbide microstructure by cooling after the earlier normalization of the heat treatment. The second set of samples, which was quenched and tempered, reached a hardness of 694 HV5. Hardening took place from a temperature of 1030 °C. The samples were tempered three times, where the samples were heated to 500 °C and the holding time at this temperature was set to one hour each. In Figure 3a,b, we can observe the resulting microstructure after quenching and triple tempering. The results show dense precipitation of carbides and tempered martensite. It is also possible to observe large primary carbides in the figures [23]. 

The second material X37CrMoV5-1 was supplied in a soft annealed condition at 780 °C throughout the cross-section and continuously cooled in the furnace. The hardness of the sample after annealing was 235 HV5. The microstructure of the base material after annealing consists uniformly distributed fine carbides in the form of spheres, presence of M_3_C (M: Fe mainly), M_23_C_6_ (M: Cr mainly), and a small ratio of M_7_C_3_ (M: V mainly), which are found in the ferritic matrix. To achieve such a microstructure in an annealed condition, special advanced metallurgical methods are carried out in all phases of steel production. This tool steel provides uniform hardness and toughness throughout the sample cross-section. This is achieved with a uniform distribution of secondary carbides in steel, even after hardening and tempering (Figure 2c,d). Samples that were hardened and tempered reached a hardness of 549 HV5. The quenching temperature was set at 1050 °C. Subsequent tempering took place in three phases at which the samples were heated to 500 °C. Holding time at this temperature was always one hour. The resulting microstructure after hardening and tempering was formed by a martensitic matrix with very small carbides with a size from 0.2 to 1 μm (Figure 3c,d). According to M.L Fares et al., carbides correspond to M_23_C_6_, where M is mainly chromium. These carbides may have both molybdenum and vanadium dissolved in them [24].

The last X45NiCrMo4 experimental material was also supplied in a soft annealed state with heating at 630 °C throughout the cross-section, which was followed by continuous cooling in an oven with a maximum hardness of 262 HV5. The microstructure of the base material is made up of ferrite grains, very fine traces of carbides, and a large proportion of lamellar perlite. The hardness of the samples after hardening and tempering was 406 HV5. The quenching temperature was set at 850 °C. Subsequent tempering took place in only one phase, where the samples were heated to 500 °C. The holding time at this temperature was also set to one hour. The microstructure after hardening and tempering was formed by a martensitic matrix with very fine spherical precipitates. The results suggested that a tempering at 500 °C provided conditions for the spheroidization of precipitates (Figure 3e,f). All heat treatments took place in a vacuum chamber on a dilatometric device DIL 805A/D (TA Instruments, New Castle, DE, USA). The samples were heated with an induction coil and cooled with nitrogen (N_2_). To determine the exact temperature, thermocouples were welded to the sample by resistance welding (TA Instruments, New Castle, DE, USA), which recorded the temperature throughout the heat treatment. All three types of tool steels have undergone high temperature tempering.

The microstructure of the basic sample was evaluated using SEM, AFM, and LOM. The samples were etched with a 3% Nital etchant. For AFM, a silicon tip with a radius of 7 nm and a spring constant of 26 N/m was used for the measurement. The microscope worked in the tapping mode, where the tip was not in constant contact with the surface, but only tapped on it to achieve longer service life to keep the tip as sharp as possible for longer measuring time, which was about 30 min to achieve the desired resolution for each figure.

### 2.1. Hardness Test

Hardness was measured using a Vickers hardness testing machine (Instron Wolpert 930) equipped with a diamond indenter under the load applied of 49.05 N and a dwell time of 10 s. The average of five indentations for each sample was taken in the results.

### 2.2. Dry Sliding Test

**Figure 4:**
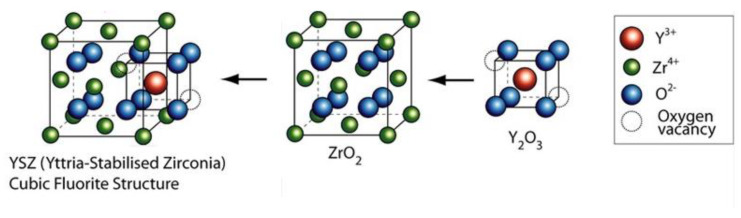
Structure Yttria-stabilized zirconia [26].

Tribological wear tests were performed using a UMT TriboLab linear reciprocating device. The configuration of the device has been set to a linear reciprocating drive module using two types of pressing materials [25]. The first material was a ceramic ball made from Zirconium oxide (ZrO_2_) used in bearings with a diameter of 4.76 mm. The ball was made by sintering ceramic powder. Zircon is stabilized by yttrium oxide with which it forms a cubic fluorite structure and, thus, achieves high hardness (Figure 4). 

The second material was a heat-treated high-alloy X46Cr13 steel ball of the same diameter. This material has good, hot workability and the potential to achieve high hardness by heat treatment. It contains 0.43% carbon, which is a good compromise between hardness and corrosion resistance, since it is alloyed with 12.5% chromium. 

**Figure 5:**
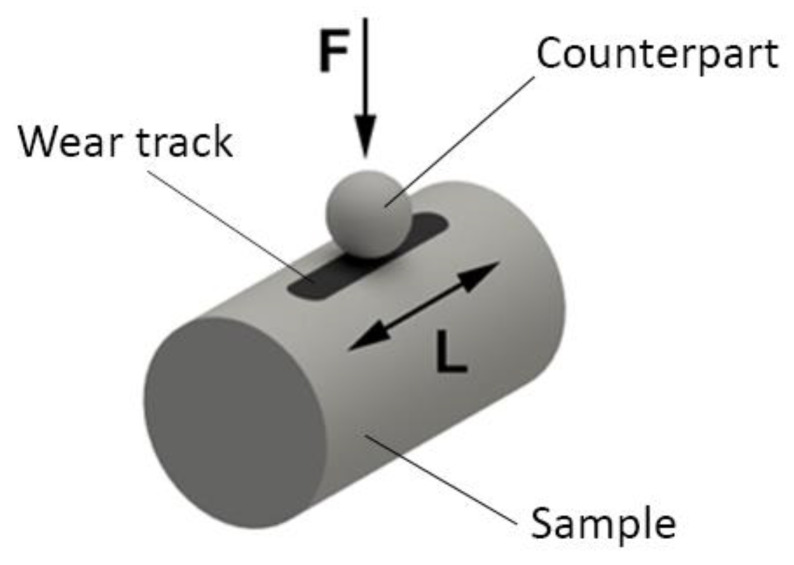
Wear measurement principle.

An example of the test, the chemical composition, and the hardness of the two counterparts are shown in Figure 5 and Table 3. Prior to each test, all ball and samples were degreased, rinsed with ethanol, and air dried. 

Linear reciprocal tests were performed at a load of 30, 60, and 90 N. The counterpart as a ball was sliding on tool steel at a rate of 5 mm/s over a 5-mm path without using lubrication. The total duration of each test was 1800 s and the total sliding distance was 9000 mm. The tool steel samples were cylindrical with a diameter of 10 mm and with the same length. The bearing balls were moved along the oval surface of a single tool steel sample. The length of the wear track was 5 mm. Table 4 summarizes the sliding conditions of the counterpart ball on the sample. All measurements were performed at room temperature. Friction coefficients were continuously registered during the test. The maximum contact area was considered for wear. Loss of material volume was calculated by multiplying the length of the friction path and by using the cross-sectional area of the scuffed area. The roughness values of balls were also measured with the AFM microscope and their average value is also shown in Table 5. 

The UMT Tribolab tribometric instrument automatically calculates and records the coefficient of friction (COF) by means of electrical sensors using the ratio of the normal force *F* and the horizontal frictional force [27].

### 2.3. Surface Roughness of Counterparts and Samples

The surface roughness of the counterparts and tool steel samples was evaluated by AFM. The measurements were carried out at five different areas. The tip with a radius of 7 nm measured the 3D topography of the surfaces. The main roughness parameter was used (arithmetical mean height (*Sa*)). This parameter expands the profile (line roughness) parameter *Ra* three dimensionally. It represents the arithmetic mean of the absolute ordinate *Z* (x, y) within the evaluation area. It provides stable results since the parameter is not significantly influenced by scratches, contamination, and measurement noise. Since a single parameter is not sufficient to adequately determine surface roughness and surface topography, other roughness parameters such as RMS roughness *Rq* (Root Mean Square), skewness *Ssk*, and kurtosis *Sku* have also been determined. According to References [28,29], skewness is the dominant parameter that affects tribological properties. The friction is lower than expected. 

**Figure 6:**
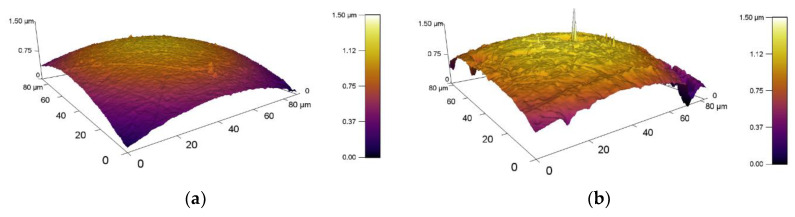
3D topography of the counterparts measured by AFM, (**a**) ZrO_2_, and (**b**) X46Cr13.

**Figure 7:**
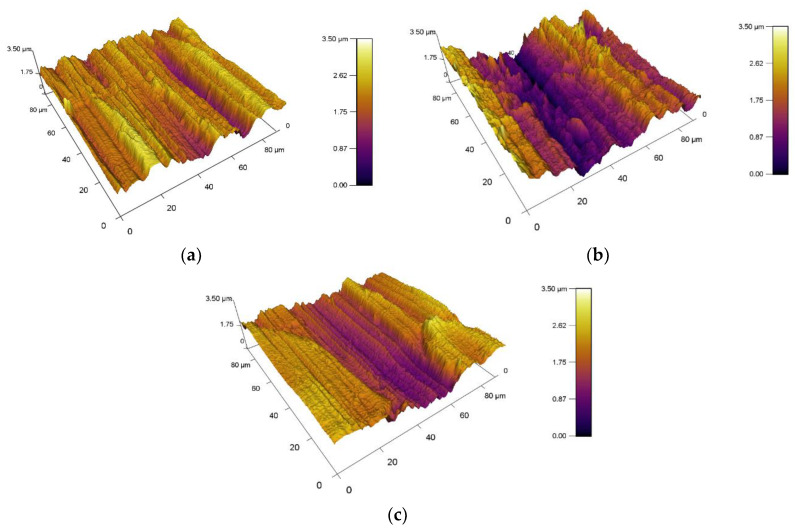
Surface roughness of experimental samples, (**a**) X153CrMoV12, (**b**) X37CrMoV5-1, and (**c**) X45NiCrMo4.

A graphical representation of the 3D topography of surface is shown in Figure 6, where the different surface roughness of the balls is clearly visible. This different result of the surface roughness is mainly due to a different kind of material as well as the manufacturing process. The surface roughness of the experimental samples is shown in Figure 7. The resulting roughness values of individual samples before tribological experiments are also listed in Table 5. Samples had similar values. Only X37CrMoV5-1 steel had a higher value where *Sa* = 501.6 nm. This higher roughness is due to the lowest hardness of the experimental material. Other roughness parameters are also very similar and, thus, their effect on the resulting tribological behavior is difficult to predict.

## 3. Results and Discussions

### 3.1. Wear Behaviour

**Figure 8:**
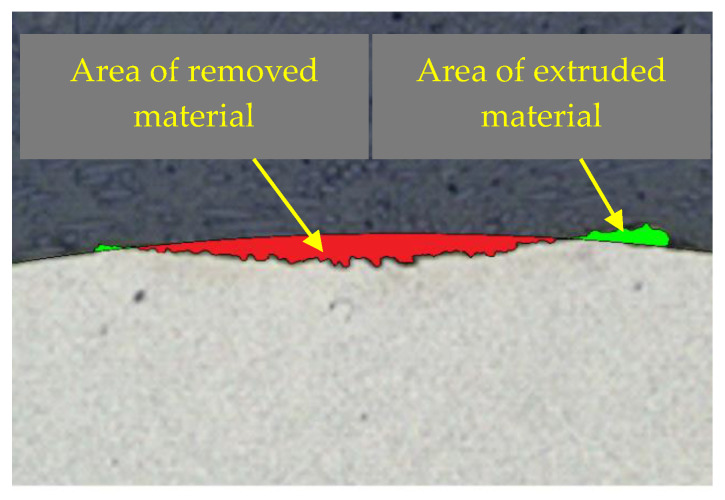
Cross-section of the wear track with marked, removed, and extruded material.

The amount of volume loss in mm^3^ was measured on samples using an optical microscope. The measured sample was cut transversely to the wear track to observing the cross-section of a formed groove. Subsequently, the area of material that was removed in the experiment was marked. In Figure 8, the area of the removed material can be seen in a red color. This area was then multiplied by the length of the wear track to calculate the overall material volume loss. In the experiment, it was shown that some of the material was not removed by friction but was extruded at the edge of the wear area. This material is illustrated by a green color. This amount of material was not considered in our case.

The volume loss of the counterpart material was negligibly low when compared to the loss of wear on the measured sample in each experiment. This minimum wear is due to the high hardness of the counterparts, which was reached for ZrO_2_ 1400 HV5 and for X46Cr13 700 HV5.

**Figure 9:**
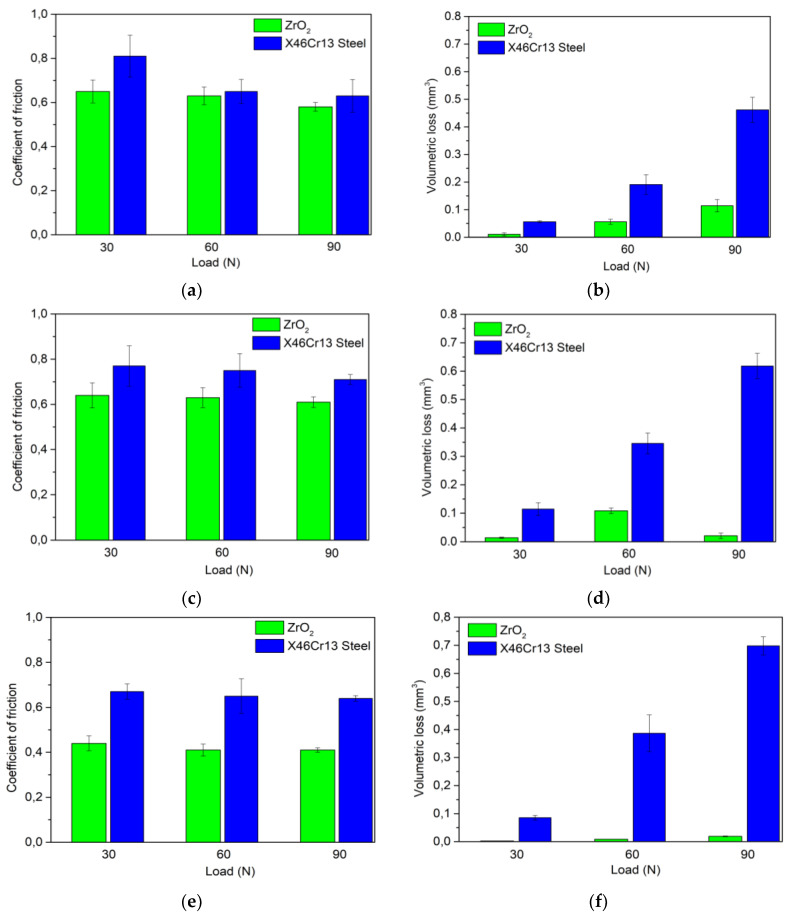
Change in friction coefficient and volume loss (annealed samples). (**a**) Coefficient of friction for material X153CrMoV12. (**b**) Volume loss for material X153CrMoV12. (**c**) Coefficient of friction for material X37CrMoV5-1. (**d**) Volume loss for X37CrMoV5-1. (**e**) Coefficient of friction for material X45NiCrMo4. (**f**) Volume loss forX45NiCrMo4.

The volume loss of selected tool steels in an annealed state is shown in Figure 9b,d,f. The results clearly show that the change in load directly affects the wear volume loss. Thus, the greater the load applied, the greater the wear of the material. This result has been demonstrated for all types of tool steels. The reduction in volume loss is attributed to the Archard’s principle in which the hardness is inversely proportional to the volume loss, which, thus, increases the hardness of the steels by decreasing the ploughing effect and reducing the volume loss [25]. The lowest volume loss can be seen in the first tool steel X153CrMoV12 (Figure 9b), which shows the best wear results only when the X46Cr13 stainless steel ball meets the experimental material. This result is due to the occurrence of very hard primary carbides M_7_C_3_ and secondary chromium carbides in the basic structure of the material. The results also show that the contact of the ZrO_2_ ceramic ball results in a significantly lower volume loss compared to the X46Cr13 steel ball. This result occurred at all loads of the material. The contact of the steel ball with the tool steel results in micro-peeling of the material, which acts as an abrasive particle and creates a cutting wedge that incises the material from the experimental sample [30]. This process affects the samples because the hardness of the bearing ball is approximately three times higher. ZrO_2_ ceramic material also exfoliates the material from the surface, but not as much as with a steel ball. For this reason, there is no large loss of material volume. 

The change in the coefficient of friction in Figure 9a indicates that the decreasing value of the coefficient is associated with an increase in the load. The same trend occurred in both types of balls. Several factors influence this result. The first is increasing the temperature between the contact materials due to an increase in the load. According to several authors [22,31], raising the temperature reduces the coefficient of friction due to the formation of a "glaze" layer consisting of sintered iron oxide wear particles. The formation of this layer, which works as a protective coating, is promoted at higher temperatures. Another factor is the interface that mediates the transmission of movement. However, it is not the geometric area that matters, but the actual area of contact, which is always smaller. The actual contact surface is dependent on the roughness of the contact bodies at which the peaks of these surfaces interact. As the loads increase, these peaks are abraded, and the contact area is increased to which the contact pressure gradually decomposes. This abrasion also generates heat and transforms it into a reduction in the coefficient of friction [32]. The resulting roughness of the base material was *Sa* = 0.34 µm, which is a very low value. In comparison to the roughness of the counterpart balls, the ball made of ceramic material ZrO_2_ was better at all three loads. This result is also due to less roughness associated with the chemical composition of the materials and their production.

The wear results of the second material X37CrMoV5-1 are similar to those of the first material. The lowest volume loss is observed at the lowest load rate of 30 N. As the load increases, the amount of volumetric wear increases proportionally to the steel ball. All three wear have a higher value when compared to X153CrMoV12. This result is due to the lower hardness of the base material after annealing. The resulting hardness is 11% lower. The results also show that the contact of the ZrO_2_ ceramic ball in comparison with the X46Cr13 steel ball results in a significantly lower volume loss. There are two types of adhesion and abrasion wear when the steel ball contacts the tool steel sample. Both types occurred on all measured samples. Paradoxically, the second contact pair of ceramic material ZrO_2_ and tool steel X37CrMoV5-1 produced the same result in which the wear rate increases as the load increases. The change in the coefficient of friction in Figure 9c indicates that the decreasing value of the coefficient of friction is associated with an increase in the measurement load. The same trend occurred in both counterparts. The factors influencing this result are the same as in X153CrMoV12. Its structural composition is associated with a decrease in the coefficient of friction because the material is formed of uniformly distributed fine carbides in the form of spheres, which are found in the ferritic matrix. This fine-grained structure also helps achieve the lowest roughness of all samples (*Sa* = 0.30 µm). The results of the last measured sample have the same character as the previous ones. The resulting wear on the ceramic ball shows the best values of all measurements. The volume loss is almost negligible (Figure 9f). This steel had a hardness of 262 HV5. By comparison, the X46Cr13 steel ball had the opposite situation. The wear rate at 90 N was the highest.

With the coefficient of friction, the situation was repeated, and the coefficient had the same nature with an increasing load (Figure 9d). Since there was a high difference between balls in the wear, there was a high difference in the coefficient of friction between the materials. This material is supplemented with nickel when compared to the other two. The nickel content significantly improves the coefficient of friction [33]. These values are best for contacting the X45NiCrMo4 experimental material with a ZrO_2_ ceramic ball.

**Figure 10:**
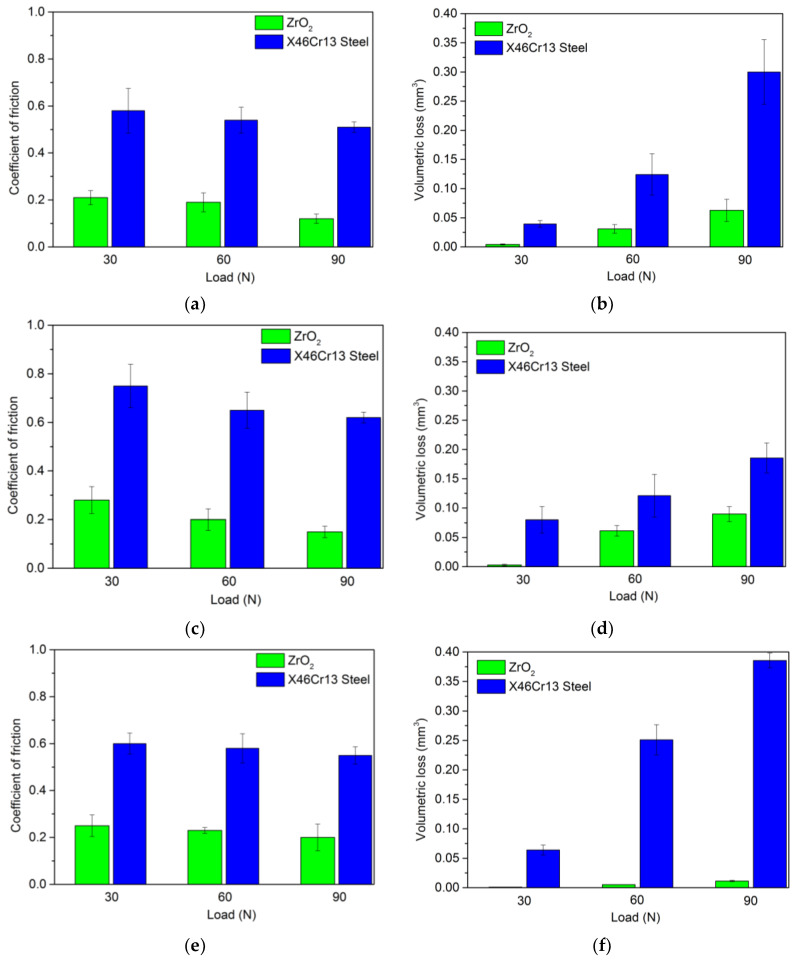
Change in friction coefficient and volume loss (hardened and tempered samples). (**a**) Coefficient of friction for material X153CrMoV12. (**b**) Volume loss for material X153CrMoV12. (**c**) Coefficient of friction for material X37CrMoV5-1. (**d**) Volume loss for X37CrMoV5-1. (**e**) Coefficient of friction for material X45NiCrMo4. (**f**) Volume loss forX45NiCrMo4.

In Figure 10b,d,f, we can observe the volumetric loss of experimental tool steels after hardening and high temperature tempering. As in the previous figure, the resulting volumetric loss values have the same character. This means that a change in load has a direct effect on the loss of volume after friction. Increasing the measurement load leads to an increase in the wear rate for all measured tool steels. Figure 10a,c,e show the change in the coefficient of friction upon contact of hardened and tempered experimental tool steels with two types of pressure balls. 

**Figure 11:**
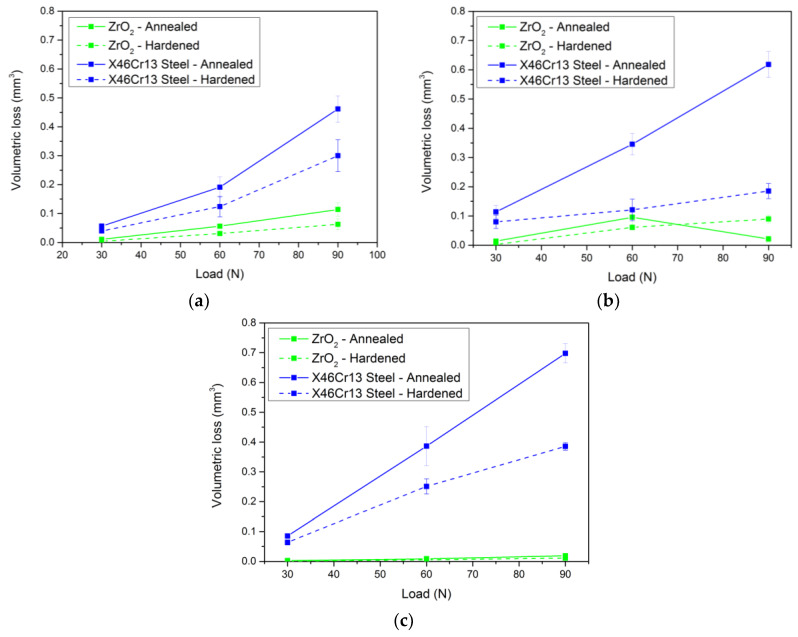
Overall comparison of the volume loss of individual tool steels in the basic-annealed and hardened-tempered state. (**a**) Volume loss X153CrMoV12. (**b**) Volume loss X37CrMoV5-1. (**c**) Volume loss X45NiCrMo4.

Figure 11 shows an overall comparison of the volume loss of individual tool steels in the basic, annealed, hardened, and tempered state. For the first examined steel X153CrMoV12, the best wear results were obtained in contact with an X46Cr13 steel ball of all experimental steels. The results show an approximate 30% decrease in the wear rate after heat treatment. Upon contact with the ZrO2 ceramic ball, there was a reduction in wear of up to 60% at a load of 30 N compared to the base annealed material (Figure 11a). After averaging the wear results, it can be stated that, in the case of the ceramic ball, the wear was reduced by almost 60% when compared to the annealed material. In the case of the steel ball, wear was reduced by approximately 35%. 

In the second investigated material X37CrMoV5-1 in Figure 11b, a significant decrease in wear due to the change in the structure of the material after hardening and tempering is observed. When in contact with the ceramic ball under a load of 30 N, the greatest reduction in wear of all measured steels was achieved (up to 80%). The average reduction in wear of this pair of materials was 53%. Contact of the X46Cr13 steel ball resulted in a comparable reduction in the overall wear rate of approximately 30% at all loads. 

The last investigated material X45NiCrMo4 showed the lowest wear rate in the basic annealed state in contact with the ZrO_2_ ceramic ball. These results were further improved by heat treatment. As in previous cases, the wear rate after hardening and tempering decreased by 65% at a load of 30 N. At additional loads of 60 and 90 N, there was a 40% decrease. On average, total wear after hardening and tempering improved by 43%. Upon contact with the X46Cr13 steel ball, the comparison of the decrease in total wear was more moderate at 30% (Figure 11c).

**Figure 12:**
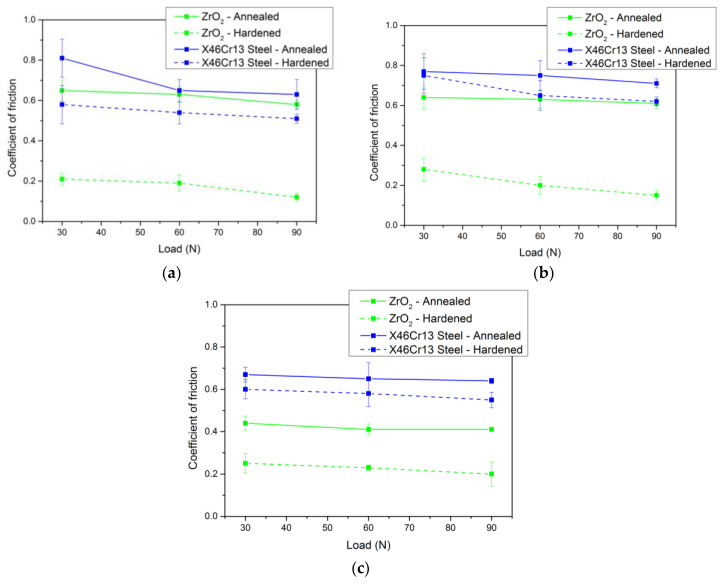
Overall comparison of friction coefficients of individual tool steels in the basic-annealed and hardened-tempered state. (**a**) Coefficient of friction X153CrMoV12. (**b**) Coefficient of friction X37CrMoV5-1. (**c**) Coefficient of friction X45NiCrMo4.

An overall comparison of all the coefficients of friction with respect to the investigated material is shown in Figure 12. The best results among all coefficients of friction after hardening and tempering were obtained with the tool steel X153CrMoV12. This steel achieved the best results both in contact with ceramic ZrO_2_ and in the X46Cr13 steel ball. Upon contact with the ceramic ball, the coefficient of friction results improved, on average, by 72%. The largest reduction in the coefficient of friction was achieved at a load of 90 N where an improvement of up to 80% was achieved (Figure 12a). Upon contact of the steel ball with the experimental material, the average value of the coefficient of friction improved by 20%.

The second investigated steel X37CrMoV5-1 achieved the second best results with the evaluated coefficient of friction with respect to the heat treatment of the given steel in contact with the ZrO_2_ ceramic ball (Figure 12b). At the highest load of 90 N, the greatest reduction in the coefficient of friction was also achieved when compared to the sample that was only annealed. The average reduction in the coefficient of friction for this pair of friction materials was 62%. The opposite results occurred with the steel ball where only an average of 10% reduction in the total coefficient of friction after hardening and tempering was achieved. 

The last investigated X45NiCrMo4 steel with respect to the final comparison of the coefficient of friction also achieved qualitative results in the contact of the ZrO_2_ ceramic ball. The average decrease in the coefficient of friction decreased by 48%, while, as with the previous two steels, the highest difference in this pair of friction materials occurred at the highest load of 90 N. Upon contact of the X46Cr13 steel ball with the given tool steel, the average value of the coefficient of friction with respect to the heat treatment was reduced by 11% (Figure 12c).

**Figure 13:**
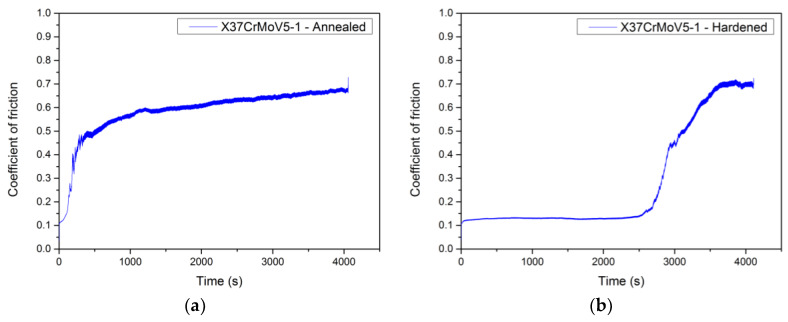
Comparison of the coefficient of friction for the material X37CrMoV5-1, ZrO_2_ ball as a counterpart, and 90 N load. (**a**) Sample in the basic annealed state. (**b**) Sample after hardening and being tempered.

In Figure 13, it is possible to observe an interesting result, where we can more deeply understand the resulting difference between the coefficients of friction before and after heat treatment. In Figure 13a, the curve of the coefficient of friction at the contact of the ceramic ball ZrO_2_ with the tool steel X153CrMoV12, which is only in the basic annealed state, is shown. In this case, we can see that the curve initially increases sharply and then equalizes to the average value of the coefficient of friction. 

In Figure 13b, the friction coefficient curve of the same contact material is shown with the only difference being that the tool steel under investigation has been hardened and tempered. Its final hardness increased significantly. In this case, we can observe that the coefficient of friction is kept constant from the beginning of the measurement (approximately 0.12) and only starts to increase rapidly at the time of about 2500 s. This increase is due to the later formation of released microparticles from the experimental material surface than in the case of the soft-annealed material. These particles begin to cut into the material and the counterpart, which increases the resistance to movement and, thus, increases the coefficient of friction. At the end of the measurement, the coefficient of friction reached similar values as in the case of soft-anealed material. The resulting hardness values after hardening and tempering play one of the most important roles in the friction process and are closely related to the value of the coefficient of friction.

### 3.2. Wear Mechanisms

**Figure 14:**
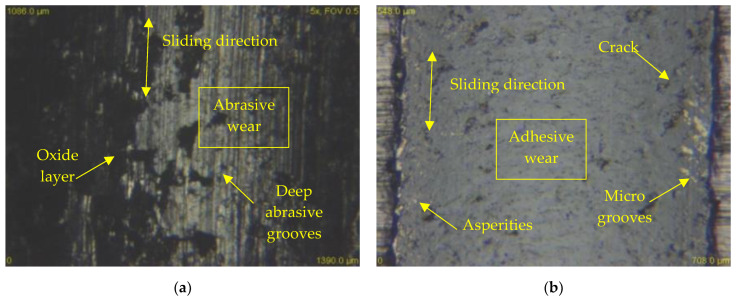
Wear analysis of worn surfaces, (**a**) X45NiCrMo4 tool steel and X46Cr13 counterpart (load 90 N), (**b**) X45NiCrMo4 tool steel and ZrO_2_ counterpart (load 90 N).

An in-depth examination of the worn surface by means of an optical microscope identifies two types of wear that occur under all load conditions and both kinds of counterpart materials. By contacting the X46Cr13 steel ball with all three types of tool steels, all three types of loads were predominantly abrasive wear with one of the intensive degradation processes of wear. The hard microparticles of the released material move freely and form cutting wedges that later create deep scratches in the materials (Figure 14a). These particles may be either loose or bound to the contact material. The released microparticles become harder than the base material due to intense plastic deformation or oxidation by air oxygen. Adhesion to this pair of metal materials occurred only to a small extent. Since the surfaces of the two bodies are never ideally smooth, the contact does not occur on the entire surface, but at many contact points. Due to the forces, the peaks on the surface are plastically deformed and the atoms of both surfaces are in close contact and form micro-bonds. These micro-couplings later break at the points above the contact of the material. This is due to the formation of a surface area, which is reinforced by plastic deformation. Their strength is higher than the strength below the surface areas. Microparticle breakage resulted in the transfer of microparticles from the surface of one body to the surface of another body. These then remained clamped to the surface of the other body or moved as loose particles between the materials to promote the formation of additional abrasive particles. 

The resulting worn grooves on the surface were exposed to air and oxidized for the X46Cr13 counterpart. As the load increased, the oxidation rate on the worn surface of two X153CrMoV12 and X37CrMoV5-1 materials also increased. Grain boundaries, dislocations, and other crystalline lattice defects significantly influence the oxidation rate and strain-induced grain refinement leads for the formation of denser and thicker oxide layers [34]. For the last material X45NiCrMo4, no visible oxidation layer was detected, which is also linked to the best friction coefficient result. In terms of volume loss, the most important role was abrasive wear. The rate of this wear was directly proportional to the increasing load, and this increasing load can also be associated with an increasing temperature that contributed to the release of microparticles and the formation of abrasive wear. The dark oxidation layers are also visible in Figure 14a.

The X46Cr13 stainless steel ball is alloyed mainly with chromium and manganese. The microstructure of the ball was formed by martensite and undissolved carbides. Manganese is used in Hadfield steels for its good wear resistance [35]. In the friction of two identical types of materials or steels, it is generally known and confirmed by the authors [36,37,38] that wear is higher than in the case of ceramic and steel contact.

At the contact of the ceramic ball ZrO_2_ with all three types of tool steel, the adhesive wear was predominant at all three types of load. The oxidation layer was less present in the ZrO_2_ counterpart than in the X46Cr13 ball. Since it was a very hard ceramic ball formed by the cubic fluorite structure, the material nearly did not release the microparticles at all. For this reason, only the adhesive wear occurred. Frictional plastic deformations occurred, and the experimental material gradually strengthened to form a hard shell that resisted volume wear. The friction groove also contained cracks and various unevenness in the form of protrusions (Figure 14b). Cracks occur predominantly in a direction perpendicular to the axis of the wear track. The resulting cracks arise after cyclic loading. The intersection of these cracks leads to the tearing of the worn part that has the shape of a thin shell. These thin shells of the material are gradually crushed and turned into hard, abrasive micro-particles.

**Figure 15:**
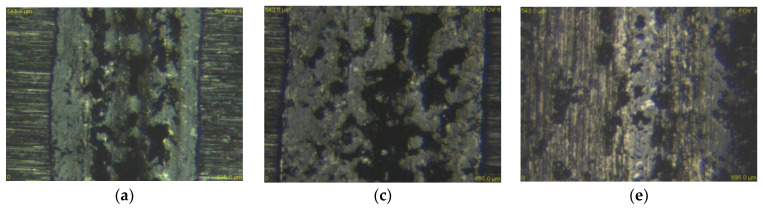

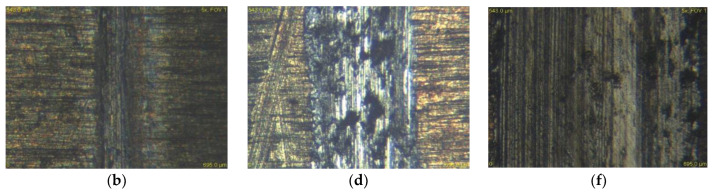
Worn surface of tribological couple X37CrMoV5-1–ZrO_2_, (**a**) 30 N—annealed, (**b**) 30 N—hardened and tempered samples, (**c**) 60 N—annealed, (**d**) 60 N—hardened and tempered samples, (**e**) 90 N—annealed, and (**f**) 90 N—hardened and tempered samples.

**Figure 16:**
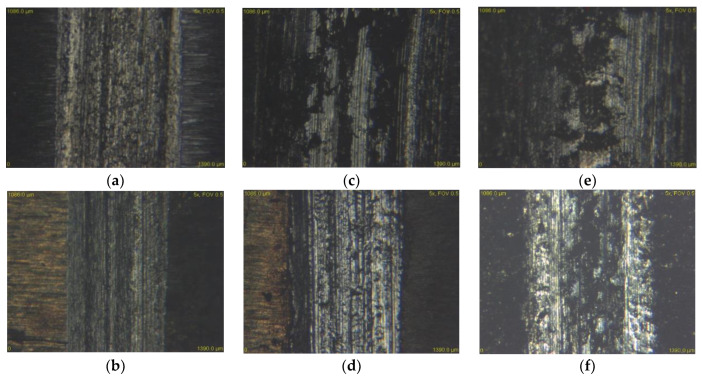
Worn surface of tribological couple X37CrMoV5-1–X46Cr13, (**a**) 30 N—annealed, (**b**) 30 N—hardened and tempered samples, (**c**) 60 N—annealed, (**d**) 60 N—hardened and tempered samples, (**e**) 90 N—annealed, (**f**) 90 N—hardened and tempered samples.

Figure 15 and Figure 16 show the experimental material X37CrMoV5-1 in contact with both types of balls and at all three loads. It is clear from the figures that the wear rate of the ceramic ball ZrO_2_ as well as the steel ball X46Cr13 decreased significantly. As already mentioned, the most significant difference in wear between the annealed and heat-treated sample occurred at a load of 30 N. These different grooves are shown in Figure 14a,b. In evaluating the wear mechanism, similar results were obtained on annealed and heat-treated samples. The only difference was the occurrence of deep scratches in the heat-treated samples. These occurred in slightly higher amounts than in the annealed samples. The reason for the higher occurrence was the higher hardness after heat treatment and, thus, the higher hardness of the released microparticles, which acted abrasively.

## 4. Conclusions

In this study, the dry sliding friction of three tool steels was investigated and their wear rates on contact with two kinds of bearing ball materials, ZrO_2_ and X46Cr13, were compared. Subsequently, the second set of samples was subjected to heat treatment in the form of hardening and tempering to a temperature of 500 °C. The evaluation also focused on the hardness, surface roughness, microstructure of the samples, and the influence of load on the resulting wear. The following conclusions can be drawn from the present work.

(1) Tool steels belonging to high strength steels can successfully represent wear-resistant steels. Because their chemical composition positively supports mechanical properties. This result has been obtained for materials, which are both in the basic annealed state and in the hardened and tempered state.
(2) Changing the contact pairs causes a significant reduction in the wear and friction coefficient. This change is associated with wear mechanisms in contact with the ZrO_2_ ceramic material. The results clearly show a reduction in wear as well as a coefficient of friction due to the predominantly adhesive wear of all samples in the basic state and the heat-treated state.
(3) Experimental steel X153CrMoV12 reached the highest hardness after heat treatment. The hardness value was 694 HV5, which represents a 250% increase when compared to the basic annealed state.
(4) The hardness of the samples also affects the resulting wear. This hardness is closely related to the microstructure of the samples. Increasing the hardness has a positive effect on the resulting wear. The experimental steel X153CrMoV12 with the presence of large primary and secondary chromium carbides achieved the least wear in contact with the X46Cr13 steel ball both in the annealed and heat-treated state.
(5) Another important factor that plays one of the most important roles is the load during tribological testing. Increasing the load results in significant degradation of the material on the sample surface. At the highest load of 90 N, the highest wear rate of all samples was also achieved. In the annealed state X37CrMoV5-1 steel and the load of 90 N, the wear value has dropped to a lower level than at the load of 60 N. The explanation for this result is to extrude the material at the edge of the friction groove. In this experiment, two types of wear, which include abrasive and adhesive wear, occurred on the surface. Under the influence of high specific load, there was a significant plastic deformation and the material was cold-welded to the edges of the groove.
(6) The roughness of the experimental samples was at a similar level and did not play a major role in wear. An important factor was the roughness of the counterparts. The ceramic ball ZrO_2_ achieved a significantly lower wear value than the steel ball X46Cr13. This is also due to the greater contact surface with the material under investigation and, thus, better distribution of the compressive load over the friction surface. Because of these small differences in skewness and kurtosis values, it is difficult to determine their effect on the resulting tribological properties.
(7) In the case of hardness, it can also be stated that the ceramic ball ZrO2 with a hardness of 1400 HV5 created a significant pressure load on all the samples and, due to the mostly adhesive wear, a plastic hardened shell was formed on the groove surface, which resisted further wear.
(8) The friction coefficient due to the increasing load had a decreasing character in all three experimental samples. This result is associated with increasing temperature due to increasing load. The increasing temperature positively affects the coefficient of friction due to the changing kinetic energy into the heat. This heat is exhibited by the vibrational movement of the atoms, which are looser and create less friction resistance.
(9) X45NiCrMo4 tool steel achieved the best wear values in contact with the ZrO_2_ ceramic ball. It achieved these results in the basic annealed state as well as in the hardening and tempering state.
(10) Lastly, it can be stated that, when comparing the coefficients of friction with respect to the heat treatment of the samples, the best improvement was achieved by the first investigated steel X153CrMoV12. The coefficient of friction improved by up to 72% on average with the ceramic ball as a counterpart. With the X46Cr13 ball as a counterpart, the improvement was 20%.

## Figures and Tables

**Table 1:** Chemical composition of investigated steels (wt.%).

**X153CrMoV12**	**C**	**Si**	**Mn**	**Cr**	**Mo**	**V**
min–max	1.45–1.60	0.10–0.40	0.20–0.60	11.00–13.00	0.70–1.00	0.70–1.00
Spectral analysis	1.53	0.35	0.40	12.00	1.00	1.00
**X37CrMoV5-1**	**C**	**Si**	**Mn**	**Cr**	**Mo**	**V**
min–max	0.33–0.41	0.8–1.2	0.25–0.5	4.8–5.5	1.1–1.5	0.3–0.5
Spectral analysis	0.37	0.88	0.35	5.10	1.35	0.40
**X45NiCrMo4**	**C**	**Si**	**Mn**	**Cr**	**Mo**	**Ni**
min–max	0.40–0.50	0.10–0.40	0.15–0.45	1.20–1.50	0.15–0.35	3.80–4.30
Spectral analysis	0.45	0.25	0.35	1.35	0.25	4.05

**Table 2:** Comparison of basic properties of tool steels.

	Hardness HV5 (Annealed)	Hardness HV5 (Hardened and Tempered)	Modulus of Elasticity [GPa]	Tensile Strength [MPa]	Yield Strength [MPa]	Elongation (%)
X153CrMoV12	270	694	210	651	447	22
X37CrMoV5-1	235	549	207	759	739	24
X45NiCrMo4	262	406	215	953	889	43

All data are for material in the annealed state.

**Table 3:** Chemical composition (wt.%), hardness, and roughness of the counterpart.

Counterpart	ZrO_2_	Y_2_O_3_	C	Mn	Si	P	S	Cr	HV5
ZrO_2_	94.80	5.20	–	–	–	–	–	–	1400
X46Cr13	–	–	0.43	0.53	0.36	0.02	0.01	12.50	700

**Table 4:** Tribology measurement parameters.

Sample	Counterpart	Load [N]	Sliding Rate [mm/s]	Sliding Length Track [mm]	Number of Repetitions [–]
X153CrMoV12	ZrO_2_	30	5	5	1000
		60			
		90			
	X46Cr13	30	5	5	1000
		60			
		90			
X37CrMoV5-1	ZrO_2_	30	5	5	1000
		60			
		90			
	X46Cr13	30	5	5	1000
		60			
		90			
X45NiCrMo4	ZrO_2_	30	5	5	1000
		60			
		90			
	X46Cr13	30	5	5	1000
		60			
		90			

**Table 5:** Roughness parameters of experimental samples and counterparts.

Material	Mean Roughness *Sa* [nm]	RMS Roughness *Sq* [nm]	Skewness *Ssk* [-]	Kurtosis *Sku* [-]
ZrO_2_ (ball)	7.8	10.7	−0.7878	12.9287
X46Cr13 (ball)	37.6	58.4	−1.8851	14.2136
X153CrMoV12	342.7	440.2	−0.3915	0.4039
X37CrMoV5-1	501.6	619.6	−0.0659	−0.6195
X45NiCrMo4	300.8	441.1	−0.4789	−0.7263
